# Stress Cardiomyopathy Managed with Extracorporeal Support after Self-Injection of Epinephrine

**DOI:** 10.1155/2017/3731069

**Published:** 2017-08-27

**Authors:** Bourenne Jeremy, Fresco Raphaëlle, Kerbaul François, Michelet Pierre, Gainnier Marc

**Affiliations:** ^1^Réanimation des Urgences et Médicale, Assistance Publique Hôpitaux de Marseille, CHU la Timone 2, Aix-Marseille Université, Marseille, France; ^2^Service d'Aide Médicale Urgente des Bouches du Rhône, CHU la Timone and UMR MD2, Aix-Marseille Université, Marseille, France; ^3^Service d'Accueil des Urgences Adultes, Assistance Publique Hôpitaux de Marseille, CHU la Timone 2, Marseille, France

## Abstract

A 28-year-old man was admitted to the ICU for self-injection of Epinephrine. This injection resulted in the rapid development of a catecholamine-induced cardiomyopathy (inverted Takotsubo) with a severe cardiogenic shock. The importance of ventricular dysfunction required the implementation of a temporary arteriovenous circulatory support until the recovery of myocardial stunning. This case allows redefining the role of circulatory assistance during cardiotropic agents intoxication.

## 1. Introduction

Stress-induced cardiomyopathy or Takotsubo cardiomyopathy is probably caused by an excess of endogenous catecholamines. Animal models suggest the occurrence of a depletion of stimulating Beta effect in a situation of catecholamines excess.

We report the case of a 28-year-old patient, admitted for an adrenergic cardiomyopathy after self-injection of Epinephrine.

## 2. Case Report

We report the case of a 28-year-old patient admitted to ICU following self-injection of 5 mg of intravenous Epinephrine. This patient presented a history of drug abuse and schizophrenia. His usual treatment included Methadone, Diazepam, Loxapine, Escitalopram, Trihexyphenidyl, Chlorpromazine, and Heptaminol. He was in-patient in a psychiatry unit under compulsatory hospitalization (on a third party request) and was discharged for the day on a permission. He consulted in the emergency department (ED) in order to obtain a benzodiazepine prescription. While alone in the emergency room, he stole intravenous Epinephrine and injected himself with 5 mg of the intravenous Epinephrine back home. He was then readmitted to the ED; he presented with intense headache with important dyspnea. His blood pressure was 80/50 mmHg and he clinically presented with an acute pulmonary edema. The transthoracic echography (TTE) showed a global hypokinesia, prevalent on the right ventricle (RV) and left ventricle (LV) bases, and a left ventricular ejection fraction (LVEF) estimated at 30% (Simpson method), with a low cardiac output (2.2 L/min). Troponin was at 2.4 *µ*mol/L and BNP 1102 ng/L. He was quickly transferred to the cardiology intensive care unit in the regional teaching hospital. He presented then with a cardiogenic shock, associating a low blood pressure at 70/40 mmHg and a sinusal tachycardia at 120 bpm. Troponin increased and reached 12 *µ*mol/l. He presented with a severe hypoxemia with polypnea (arterial blood gazes showed a PaO2 50 mmHg, arterial lactate 2.6 mmol/l, and a SpO2 at 85% in ambient air). His urine output was however normal at the time (100 mL/h). Chest X-ray showed a bilateral interstitial syndrome beginning at the hilum of the lungs. Electrocardiogram presented no troubles of ventricular repolarization.

The global hypokinesia predominated in the basal regions of the heart, excluding the cardiac apex, with a persistent severe global systolic dysfunction on TTE; the LVEF was evaluated at 25% and the subaortic velocity time integral was 13 cm/s under Dobutamine 7 *µ*g/kg/min.

The diagnostic hypothesis was an adrenergic cardiomyopathy with intact coronary arteries such as reverse Takotsubo cardiomyopathy, responsible for a cardiogenic shock.

The evolution was marked by the occurrence of syncope with seizures associated with a major hypotension (50/30 mmHg). He was then transferred to ICU in this context of vigilance troubles related to a low cardiac output. The patient was intubated and placed under mechanical ventilation, sedated by Midazolam and Sufentanil. Hemodynamics parameters were very unstable despite the high doses of exogenous catecholamine administrated (Dobutamine 20 *µ*g/kg/min, Epinephrine 0.4 *µ*g/kg/min, and Norepinephrine 1.1 *µ*g/kg/min). We did not retain the coronary occlusion hypothesis because of a lack of arguments and a decreasing Troponin level at 6 *µ*g/l and we did not performed a coronarography for these reasons. A bilateral areactive mydriasis was observed at the 14th hour of care in ICU, whereas, before induction and intubation, pupils were intermediate and reactive. Transcranial Doppler showed a normal arterial flow in the Middle Cerebral Artery. Systolic dysfunction increased and LVEF lowered until 10%.

An arteriovenous extracorporeal circulatory assistance (ECLS) is initiated at day 1 (assistance output 4.5 L/min, 3500 rpm).

Considering the bilateral mydriasis, a cerebral CT-scan is performed under extracorporeal assistance and revealed to be normal. The thoracic angioscan performed in the meantime showed an inferior right lobar pneumonia and a bilateral proximal pulmonary embolism. A biantibiotherapy with Ticarcillin-Clavulanic Acid and Gentamicin was introduced as the patient presented with a fever at 40°C. We identified a Methicillin-Sensitive* Staphylococcus aureus* in bronchial sample and the antibiotherapy was reduced to Oxacillin.

At day 1, Epinephrine has been stopped and at day 3, the echocardiography found an improvement of apical contractility, allowing a progressive reduction in the assistance output (2000 rpm) with an excellent tolerance of the LV and an adequate organ perfusion.

Weaning from assistance was pursued under Dobutamine and Norepinephrine. At day 5, the patient was completely weaned and the cannula was removed. The Troponin was normalized.

Systolic function has progressively reached a normal range (LVEF 40% under 20 *µ*g/kg/min of Dobutamine). Dobutamine is gradually reduced and Norepinephrine was stopped after an introduction of a substitutive hormonotherapy with Hydrocortisone.

Sedation was interrupted at day 5 and the patient was rapidly awake without delirium. Extubation was performed at day 8 with a relay with noninvasive ventilation and nasal high flow oxygen therapy due to a persistent hypoxemia.

After rehabilitation started in ICU, the patient was discharged without sequel at day 17. At one year, he is still hospitalized in a psychiatry unit.

## 3. Discussion

Our observation is the first case reporting the institution of a peripheric extracorporeal assistance in a transitory stress-induced cardiomyopathy due to self-injection of Epinephrine. In the literature, there is only one description; the patient presented after an injection of 2 mg of Epinephrine with a transitory LV dysfunction with apical ballooning evoking an adrenergic cardiomyopathy. In addition to vasopressors agents, the treatment needed to be implemented with an intra-aortic balloon pump [1]. We found other observations which describe the utilization of extracorporeal circulatory assistance for Takotsubo cardiomyopathy with favorable evolution. Takotsubo is a reversal etiology of cardiogenic shock; it can be caused by endogenous secretion of catecholamine as a pheochromocytoma [2]. Bonacchi et al. described a series of four severe polytraumatized patients with inverted Takotsubo cardiomyopathy; all patients were treated by ECLS with a favorable evolution for 2 patients. For 2 patients, posttraumatic cerebral death occurred and they underwent organ explanation [3].

Takotsubo cardiomyopathies have been described in case of Epinephrine injection in anaphylactic reactions [4, 5] but none has ever needed an extracorporeal assistance. Takotsubo cardiomyopathy is characterized by a disharmonious hypokinetic cardiopathy, with in its first description an apical ballooning [6] caused by a hypokinesia of the medioapical region and a normal contractility of basal regions. Since this first description, several clinical presentations have been described with different myocardial regions affected [7]: hypokinesia of apical and medial region is the most frequent dysfunctions in 60% of the cases. Yet the dysfunctions of medial regions (named as medioventricular forms) or isolated basal regions (reverse or inverted Takotsubo) have been described. In our case report, hypokinesia was global and responsible for a refractory cardiogenic shock.

Several pathophysiological hypotheses can explain this acute cardiomyopathy, among them the massive release of endogenous catecholamines due to a hyperactivation of the sympathetic system caused by an external stress. In a study including 19 patients affected with Takotsubo cardiomyopathy, Wittstein et al. found endogenous catecholamines levels 2 to 3 times higher than those in patients with acute coronary syndrome and 7 to 34 times higher than the physiological level [8].

The myocardial dysfunction in the precise medial and apical regions is due to the gradual repartition of Beta adrenergic receptors in the myocardial muscle. Indeed, apical and medial regions express predominantly negative inotropic Beta 2 adrenergic receptors, whereas basal regions express a majority of positive inotropic Beta 1 adrenergic receptors [9].

The excess of endogenous or exogenous amines is responsible for a myocardial toxicity related to an important stimulation of Beta receptors leading to a hyperactivation of proteins G coupled to the receptors behind a raise in intracytosolic Calcium concentration [10, 11]. The reduced stock in Calcium in the sarcoplasmic reticulum leads to the abnormal contractility observed. In the meantime, a downregulation of Beta receptors occurs due to their hyperactivation with an internalization of the receptors. This phenomenon explains the lack of sensibility of these patients to Beta vasopressors agents that may be introduced as a therapeutic [12]. This case report confirms the hypothesis that Takotsubo cardiomyopathy is caused by an excess of catecholamines whether they are endogenous or exogenous.

A recent review on therapy of stress cardiomyopathy [13] recommended, after fluid resuscitation, the cessation of inotropic therapy and infusion of Beta blocker. Despite the clear evidence that administration of catecholamine may precipitate the apical ballooning, we use high dosage of Norepinephrine and Dobutamine. Reverse effect of Beta blocker therapy in stress cardiomyopathy is not so evident and is not intuitive in the treatment of cardiogenic shock.

Nowadays, extracorporeal circulatory assistance has a great importance in cardiotropic agents intoxication management. The mortality induced by these intoxications is principally related to the occurrence of a multiorgan failure caused by a refractory cardiogenic shock [14]. The implementation of an extracorporeal circulatory assistance is realized by femorofemoral cannulation. Circulatory assistance allows restoring a sufficient organ perfusion output to avoid the hypoperfusion vicious circle, responsible for repeated ischemia and reperfusion syndromes, leading to multiorgan failure [15]. Despite the absence of high level of scientific proofs, the indication of circulatory assistance is justified by the temporary effect of the toxic agent and by the fact that a complete recovery of the left systolic function is expected after toxic elimination. The expected duration of cannulation is generally short, which allows avoiding the mechanical complications related to extracorporeal circuits. The survival of the patients depends on the precocity of establishment of the assistance, before the occurrence of irreversible organs failure [16].

An early implementation of circulatory assistance allowed in this case report a rapid weaning without complications related to the technique.

## 4. Conclusion

Epinephrine self-injection created in this patient an experimental model of transitory stress-induced cardiomyopathy and confirmed the adrenergic hypothesis of this syndrome. The implementation of the circulatory assistance allowed in this case restoring an efficient cardiac output before the occurrence of a multiorgan failure.
