# Corrigendum to “Stress Cardiomyopathy Managed with Extracorporeal Support after Self-Injection of Epinephrine”

**DOI:** 10.1155/2018/3121408

**Published:** 2018-08-09

**Authors:** Jeremy Bourenne, Raphaëlle Fresco, François Kerbaul, Pierre Michelet, Marc Gainnier

**Affiliations:** ^1^Réanimation des Urgences et Médicale, Assistance Publique Hôpitaux de Marseille, CHU la Timone 2, Aix-Marseille Université, Marseille, France; ^2^Service d'Aide Médicale Urgente des Bouches du Rhône, CHU la Timone and UMR MD2, Aix-Marseille Université, Marseille, France; ^3^Service d'Accueil des Urgences Adultes, Assistance Publique Hôpitaux de Marseille, CHU la Timone 2, Marseille, France

In the article titled “Stress Cardiomyopathy Managed with Extracorporeal Support after Self-Injection of Epinephrine” [1], the authors' names were reversed. The revised authors' list is shown above.
